# Schistosomiasis transmission and environmental change: a spatio-temporal analysis in Porto de Galinhas, Pernambuco - Brazil

**DOI:** 10.1186/1476-072X-11-51

**Published:** 2012-11-20

**Authors:** Elainne Christine de Souza Gomes, Onicio Batista Leal-Neto, Jones Albuquerque, Hernande Pereira da Silva, Constança Simões Barbosa

**Affiliations:** 1 Laboratory of Parasitology, Vitoria Academic Center, Federal University of Pernambuco, Recife, Pernambuco, Brazil; 2 Schistosomiasis Laboratory and Reference Service, Department of Parasitology, Aggeu Magalhães Research Center, Fiocruz, Recife, Pernambuco, Brazil; 3 Department of Statistics and Computing, Federal Rural University of Pernambuco, Recife, Pernambuco, Brazil; 4 Geosere - Laboratory of GIS and Remote Sensing, Department of Rural Tecnology, Federal Rural University of Pernambuco, Recife, Pernambuco, Brazil

**Keywords:** Schistosomiasis, Remote sensing, Epidemiology, Spatial analysis

## Abstract

**Background:**

In Brazil, schistosomiasis mansoni infection is an endemic disease that mainly affects the country’s rural populations who carry out domestic and social activities in rivers and water accumulations that provide shelter for the snails of the disease. The process of rural migration to urban centers and the disorderly occupation of natural environments by these populations from endemic areas have favored expansion of schistosomiasis to locations that had been considered to be disease-free. Based on environmental changes that have occurred in consequent to an occupation and urbanization process in the locality of Porto de Galinhas, the present study sought to identify the relationship between those chances, measure by remote-sensing techniques, and establish a new endemic area for schistosomiasis on the coast of Pernambuco State - Brazil.

**Methods:**

To gather prevalence data, two parasitological census surveys were conducted (2000 and 2010) using the Kato-Katz technique. Two malacological surveys were also conducted in the same years in order to define the density and infection rate of the intermediate host. Based on these data, spatial analyses were done, resulting in maps of the risk of disease transmission. To ascertain the environmental changes that have occurred at the locality, images from the QuickBird satellite were analyzed, thus resulting in land use maps.

**Results:**

Over this 10-year period, the foci of schistosomiasis became more concentrated in the Salinas district. This area was considered to be at the greatest risk of schistosomiasis transmission and had the highest prevalence rates over this period. The study illustrated that this was the area most affected by the environmental changes resulting from the disorderly urbanization process, which gave rise to unsanitary environments that favored the establishment and maintenance of foci of schistosomiasis transmission, thereby consolidating the process of expansion and endemization of this parasitosis.

## Introduction

In Brazil, around 30 million people are exposed to the risk of contracting schistosomiasis, and it is estimated that 4–6 million individuals are infected with *S. mansoni*[1,2]. The northeastern region of the country is markedly the most endemic area [3], and for many years, schistosomiasis was considered to be a characteristic ailment of rural areas [4-6]. However, over recent decades, there has been increasing incidence of cases in urban and coastal areas [7-14]. One of the factors noted in this process of expansion of schistosomiasis is the migratory flow of the infected rural population, which, attracted by employment opportunities in urban and coastal localities, has ended up becoming established in peripheral agglomerations where the lack of sanitation and basic infrastructure result in fecal contamination of aquatic environments, with consequent infection of intermediate host and emergence of new foci of schistosomiasis transmission [8,14].

Within this perspective, it is crucial to understand the environmental factors that give rise to occurrences of schistosomiasis in coastal areas, in order to identify the mechanisms for transmission and maintenance of this endemic disease. For this, remote sensing is becoming a valuable tool for identifying environmental changes that may directly or indirectly influence on occurrences of a disease. Studies using this technique have been shown to be effective for predicting the risk of infection, thereby enabling greater completeness of understanding about the large-scale ecology and distribution patterns of schistosomiasis, and also highlighting the influence of the determining factors within the microenvironment (parasite and snail) relating to transmission of this disease [15]. Environmental changes can be viewed and measured using this technique, thus indicating that controlling the transmission and monitoring the areas at risk can be backed up through knowledge of changes to the plant coverage, topography and water courses, from before to after episode of flooding, by means of spatial models of geographical information systems and remote sensing [16].

The urbanization process has also been highlighted as a determining factor for occurrences of helminthiasis, given that this has a direct impact on the environment. Disorderly occupation of peripheral areas is characterized by overcrowding in improvised constructions and inadequate sanitary conditions, and this may be associated with increased transmission of helminthiasis [17] and schistosomiasis [18]. These authors pointed out that the risk of infection was higher for immigrants coming from non-endemic areas, thus emphasizing the need for studies on the impact of the urbanization process on schistosomiasis and their importance for understanding the factors that have led to expansion of this disease. In this regard, it has been estimated that by 2030, around 60% of the world’s population will be living in cities and that 93% of urban growth will be occurring in developing countries [19].

This urbanization process can be detected and the environmental degradation, defined in this paper as any change or disturbance to the natural environment, as for example: reduction of mangrove and vegetation areas, can be analyzed through remote-sensing techniques. These make it possible to measure the changes in occupation of natural areas over the course of time, by using data such as normalized difference vegetation index (NDVI), thermal index, digital elevation model (DEM) and using of land, from satellites image. Together with Geographic Information System (GIS), satellite image data have been used to correlating the transmission of schistosomiasis in China [16,20] by analyzing the hydrographical transportation of intermediate host snails, and also the impact of irrigation system and seasonal flood on snail dispersal and the transmission of schistosomiasis. In Africa, satellite data have been used for surrogates of climate data in the development of environmental risk models for schistosomiasis [21], and in Brazil, similar studies were conducted by using GIS and remote sensing techniques to evaluate the risk of schistosomiasis and also to establish a statistical model for estimating its prevalence [22-24], which demonstrates the importance of those methods to evaluate the transmission and maintaining this ailment.

In Brazil, other studies have shown relationships between occurrences of schistosomiasis and environmental factors, highlighting the patterns of contact between at-risk populations and contaminated water accumulations [7,25]. It was also reported the relation between snail breeding sites and the unorganized urbanization process, where no sanitation and stormwater drainage provide all conditions to the increase of temporary breeding sites of *B. glabrata*[26]. However, models for estimating the transmission risk from temporal and spatial perspective are necessary in order to construct future scenarios in which the planning for healthcare action might be more effective, thereby minimizing the transmission and damage to health among individuals exposed to risk of this parasitosis.

The present study sought to identify environmental changes that have taken place as consequences of the process of occupation and urbanization of a locality, where the population increase was observed through the increase of housing construction from 2000 to 2010. However, this increase was not followed by sanitary infrastructure of households, which characterizes a disorderly occupation of space [26]. Therefore, by means of spatial-temporal analysis based on satellite images and malacological and epidemiological data we sought to correlate these changes with the process of expansion and endemization of schistosomiasis in a coastal region of Brazil.

## Materials and methods

**Figure 1:**
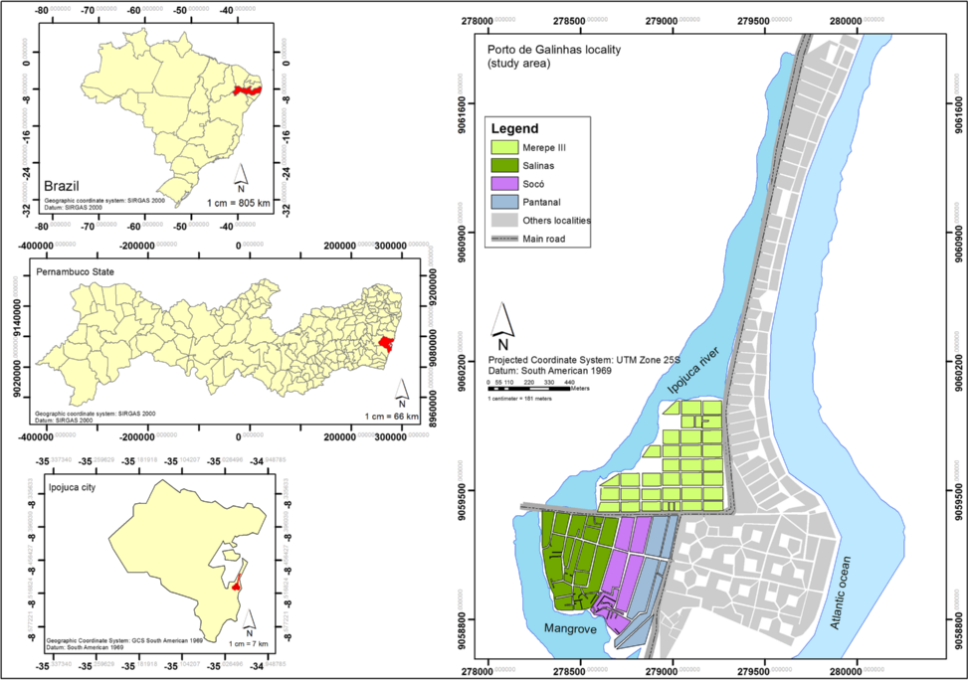
**Map of Porto de Galinhas**, **Ipojuca**, **Pernambuco state** - **Brazil.**

This study was developed in the locality of Porto de Galinhas, in the municipality of Ipojuca, on the southern coast of the state of Pernambuco - Brazil, at a distance of around 60 km from the state capital. The study area was composed by the districts of Merepe III, Salinas, Socó and Pantanal (Figure 1). Porto de Galinhas was chosen because of reports of breeding sites and foci of the snail (*Biomphalaria glabrata*) and the high numbers of schistosomiasis cases that have been notified by the Schistosomiasis Reference Service and Laboratory of the Aggeu Magalhães Research Center, Fiocruz (CPqAM-Fiocruz), over the last ten years. For this present paper, secondary data results of epidemiological surveys (malacological and prevalence studies) conducted in Porto de Galinhas in 2000 [7,11], and primary data results of the epidemiological survey conducted in 2010 [26].

### Data gathering and analysis

The data gatrering and analysis will be introduced in the next subchapters. For better understanding of the methodology adopted, this section was divided into methods of mapping, malacological survey, prevalence survey, spatial data analysis and remote sensing.

### Mapping of the locality

The locality was georeferenced by means of the global positioning system (GPS) technology, using a GPS receiver with minimum accuracy of 10 meters, configured in the Universal Transverse Mercator (UTM) projection Datum SAD 69. The locality was mapped on ground by walking, and the unit of mapping was the block. Subsequently, using the TrackMaker Pro software, the GPS receiver data was transferred to a desktop computer, in which the data were processed, thus making it possible to save files (map of the locality, case distribution, breeding sites and foci) in format that were used in the spatial data analysis, which was done by means of the ArcGis software, versions 9.3 and 10.

### Malacological survey

All the breeding sites of *B. glabrata* that were found in the study area were identified and georeferenced. Subsequently, snails were collected from each breeding site using scoops and tweezers for 15 minutes and were properly packed into moistened ventilated plastic tubs, with labeling, for transportation to the Schistosomiasis Reference Service of CPqAM//Fiocruz. At the laboratory, the infection was diagnosed and the foci of disease transmission were identified (i.e. breeding sites of *B. glabrata* in which infected snails were identified), by means of the technique of exposure to artificial light for cercariae to emerge [27]. Negative snails were reexamined 15 days later, and those that remained negative were squashed on glass plates measuring 15 x 9 cm and were examined individually to search for sporocysts (larval stages) of *S. mansoni*[28]. To determine the snails’ natural infection rate, the proportion of the mollusks that were positive for *S. mansoni* in relation to the total number of mollusks examined was calculated. The foci of schistosomiasis transmission were taken to be the breeding site that presented infected snails.

### Prevalence survey

Two stool examination census surveys were conducted (2000 and 2010) among all individuals living in the study area by using the same methodology: all individuals were asked to register and participate voluntarily; after signing the consent form given by the ethics committee, it was given a plastic container to collect stool sample, identified by the name of each participant that agree in being part of this research. The next day stool samples were collected and sent to the laboratory to be examined [26]. It was signed up 2,830 and 5,607 dwellers for the respective years 2000 and 2010. The number of the sample size was highest than the sample size calculated, it also was proportional and representative for the study localities because around 50% of the dwellers from each study site were sampled, demonstrating the representativeness of the analyzed population. The age and gender of sampled population it was proportionally represented in both years 2000 and 2010, being schistosomiasis more prevalent in teenagers and young adults (70% together) and also in male sex (around 60%). To diagnose the number of cases of schistosomiasis and the parasitic load of the parasitized individuals, feces samples were parasitologically diagnosed by means of the Kato-Katz method [29], with one sample from each participant, from which two slides were examined.

The prevalence of schistosomiasis in the blocks and localities of Porto de Galinhas was defined from the results of the parasitological examination on the feces, where prevalence rate was defined as the number of positive individuals per block or locality per total number of individuals examined per block or locality and multiplied by 100.

The χ2 test was used to compare the prevalences between the years 2000 and 2010, by means of the EpiTable in the EpiInfo 6.04 software, from which the statistical significance of this variation was obtained.

### Spatial data analysis

The malacological and epidemiological data were used to define risk areas using kernel density analysis for points (kernel intensity estimator). For malacological data analysis was considerate the board of breeding sites of *B. glabrata* and for epidemiological analysis was used the centroid of the street block. Kernel density analysis is a non-parametric way to estimate the probability density function of a random variable, by calculating the density of point features around each output raster cell. Through this method is possible to identify hot spots of the subject studied, which in this study represent risk areas to schistosomiasis transmission. The parameters used to kernel analysis were: method data classification "Quantil"; and bandwidth method defined using an adaptive beam, which is more appropriate for analyzing local studies [30,31]. This was established as 230 meters for analyses on the malacological data (breeding sites and foci of *B. glabrata*) and 200 meters for the data per street block from the parasitological survey. These parameters were defined by Araújo [7], with the area unit defined in m^2^.

### Remote sensing

To investigate the impact of the environmental changes that occurred over a four-year period, with regard to the spatial and dynamic risk of schistosomiasis transmission, satellite images with high spatial resolution were requested in order to illustrate the period of August 2006 and June 2010 analyzed in this paper. These images were generated by means of panchromatic (PAN) and multispectral (MS) sensors on board the QuickBird satellite, with spatial resolution of 0.6 and 0.5 meters, respectively. It should be noted that this study could only be conducted over a four-year period, because of the lack of high-resolution images for years preceding 2006.

To analyze the images, the ArcGis 9.3 software was used. In this, seven polygonal information planes were created using the Arccatalog tool, and the following thematic classes were associated with them: water, buildings, mangrove swamp pools, impermeable soil, permeable soil and vegetation. After the vector files had been created, the images were classified visually such that the thematic polygons were identified and edited at a scale of 1:500. Error in interpreting the images was minimized by having the two images classified by the same person. Those images were classified in these categories because they were related to a consequence of the process of urbanization over environment.

The aim in this classification was to construct thematic maps of land use and to calculate the areas of these thematic classes and by that to identify the percentage change in areas with on vegetation and in urban agglomeration between 2006 and 2010.

Comparison of the information extracted from the 2006 and 2010 images generated indicators of geographical space occupation by the human population, and of degradation of the natural environment due to the accelerated process of disorderly urbanization. The indicators of geographical space occupation used in this work are: reduction in mangrove area, in permeable soil area, reduced vegetation cover and reduced paired water (ground). Increase of built area and impermeable soil (mainly occurring in the paving of access roads).

To delimit the study area, the urban limits on the most recent image were used, enabling comparison of the same area in 2006 and 2010. This comparison according to class in these two years was done using the Microsoft Excel 2007 and EpiInfo 3.5.3 software.

## Results and discussion

### Malacology

The analyses resulting from this study made it possible to delineate the schistosomiasis transmission profile in the locality of Porto de Galinhas over the last ten years.

**Figure 2:**
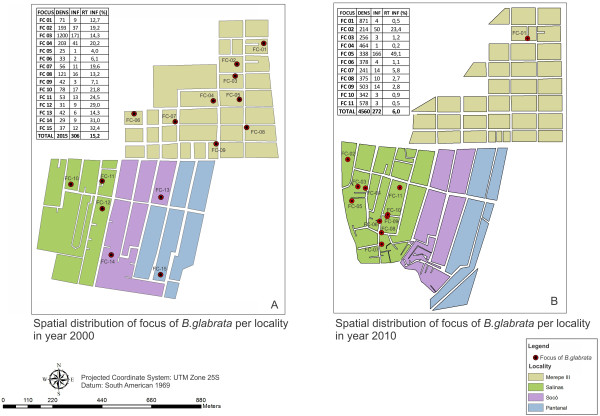
**Map of the distribution of *B****.****glabrata *foci in 2000 and 2010**, **Porto de Galinhas**, **Pernambuco - Brazil.**

The snails *B. glabrata* can be considered to be a good biological indicator for assessing the schistosomiasis transmission process, given that analysis on the spatial dynamics of these mollusks provided a closer estimate of the chances of becoming infected by *S. mansoni*. Figure 2 presents the distribution of the schistosomiasis transmission foci for the years 2000 and 2010 with the population density data and the infection rate per focus. In 2000, the foci were distributed as: Merepe III (9), Salinas (3), Socó (2) and Pantanal (1). In that year, the general infection rate was 15.2%, ranging from 4 to 32.4%. The infection rate per focus varied around the mean, and demonstrated homogenous rates between breeding sites and consequently, in space. In 2010, the number of foci decreased to 11, with a notable concentration of 10 foci in Salinas, whereas Merepe III, with nine foci in 2000, went down to just one in 2010. It was also observed that the overall infection rate decreased to less than half (from 15.2 to 6.0%) and that the variability of this rate between the foci broadened to 0.2 – 49.1% (Figure 2B).

**Figure 3:**
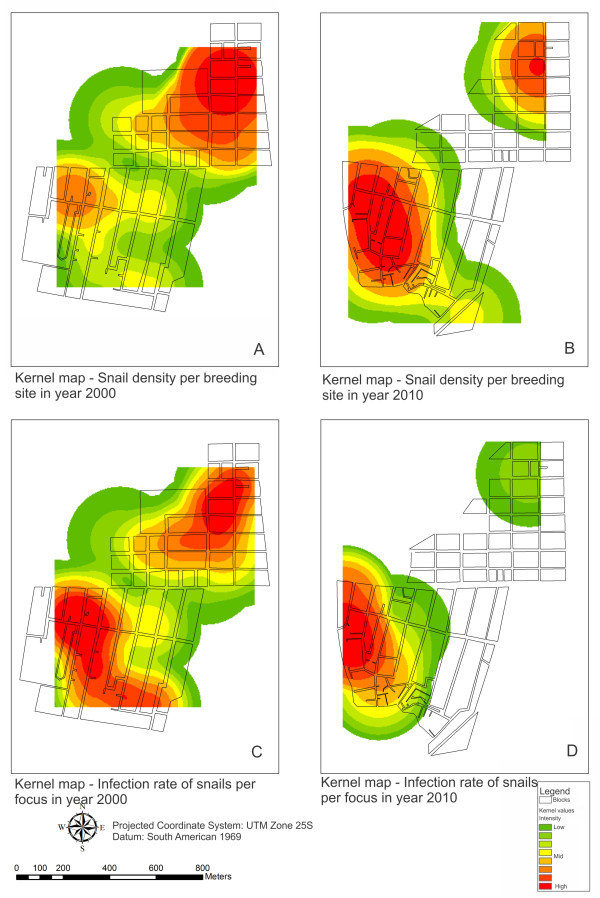
**Kernel map of snail density and infection rate per transmission focus in 2000 and 2010**, **Porto de Galinhas**, **Pernambuco - Brazil.**

Figure 3 presents the displacement of the area at risk over time, in relation to the density and infection rate of the snails in the different districts of Porto de Galinhas. With regard to density, it can be seen from the coloring on Map A that the area at risk was concentrated in the Merepe III district in 2000, and that, despite the decreased number of foci in 2010, this district still presented a high snail density, represented by the colored area at risk in the northern part of this locality (Map B). In 2010 (Map B), the area at highest risk was in Salinas, as expected. Regarding the distribution of areas at risk in relation to the infection rate in 2000, two large colored areas could be seen: one in Merepe III and another extending from Salinas to Pantanal (Map C). In relation to 2010, the area at risk of schistosomiasis transmission was restricted to Salinas (Map D).

The locality of Salinas, which currently contains most of the foci of schistosomiasis infection, is the poorest and most populous district of Porto de Galinhas. This district is home to the neediest segment of the population, living under precarious conditions of basic and environmental sanitation, with sewage outflow in the open air, unpaved streets and lack of rainwater drainage system. This scenario provides ideal conditions for maintaining breeding sites and concentrations of mollusks. In association with these factors, the proximity of this district to the mangrove swamps makes it vulnerable to periodic flooding caused by overflows from the Ipojuca river, which borders this locality. The floods carry the fecal material (exposed in the streets) and the infected snails to other localities, favoring the emergence of new foci of transmission, exposing residents and vacationers in other districts to the risk of contracting this disease. The kernel maps (Figure 3C and 3D) show that this locality presented the highest risk of schistosomiasis transmission with regard to the chances of contact with the transmission foci.

### Prevalence of schistosomiasis

The prevalence surveys conducted in 2000 and 2010 sampled 2,012 and 2,459 individuals, respectively. In these years, 653 and 409 cases of schistosomiasis were diagnosed, it illustrated that between these years, there was a reduction of around 50% in the prevalence of this disease at this locality (Table 1). Merepe III was the locality that presented the greatest reduction in prevalence, declining from 29.4 to 4.4. With the exception of Salinas, all the other localities presented decreases of at least 50%.

**Table 1:** **Prevalence of schistosomiasis in Porto de Galinhas**, **Pernambuco** - **Brazil in 2000 and 2010**

**Districts**	**Surveyed(*n*)**		**Positives(*n*)*S. mansoni***		**Prevalence(%)**	
	**2000**	**2010**	**2000**	**2010**	**2000**	**2010**
Merepe III	292	315	86	14	29.4	4.4
Salinas*	771	1,263	169	259	21.9*	20.6*
Socó	462	590	157	96	33.9	16.3
Pantanal	487	291	241	40	49.5	13.7
Total	2,012	2,459	653	409	32.4	16.6

* p-value > 0.05 (i.e. without any statistically significant difference) – χ2 test.

**Figure 4:**
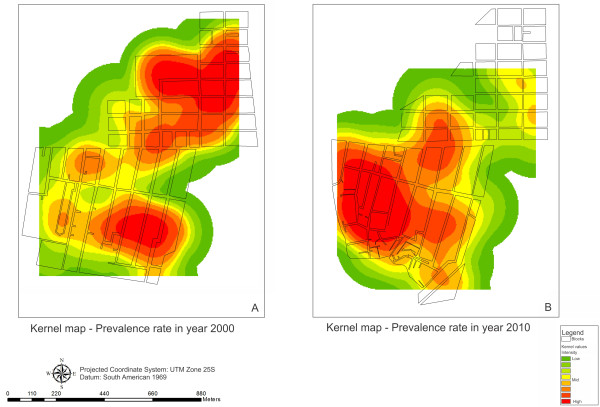
**Kernel map of schistosomiasis prevalence per street block**, **for 2000 and 2010,Porto de Galinhas, Pernambuco - Brazil.**

Figure 4 presents the kernel prevalence maps of cases per street block for the years studied. From this, the areas at highest risk of occurrences of schistosomiasis can be identified and the spatial changes in risk between 2000 and 2010 can be compared. On these maps, the shifts in the risk of occurrences of schistosomiasis among the resident population can be seen. In 2000, Merepe III presented a colored area of risk (red color) that almost completely covered this area. Pantanal was the area presenting the second highest risk, with a more limited colored area, going as far as the boundary with Socó. In that year, Salinas was the district with the lowest risk of occurrences of cases. This risk situation was found to have completely changed ten years later, when Salinas became the area with the highest chance of occurrences of schistosomiasis, with an area at risk that also reached parts of Socó and Pantanal.

**Figure 5:**
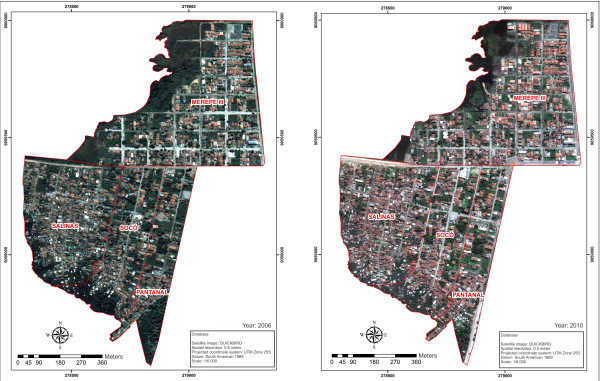
**Satellite Image map of the area of epidemiological importance for schistosomiasis in Porto de Galinhas, Pernambuco - Brazil, 2006–2010.**

**Figure 6:**
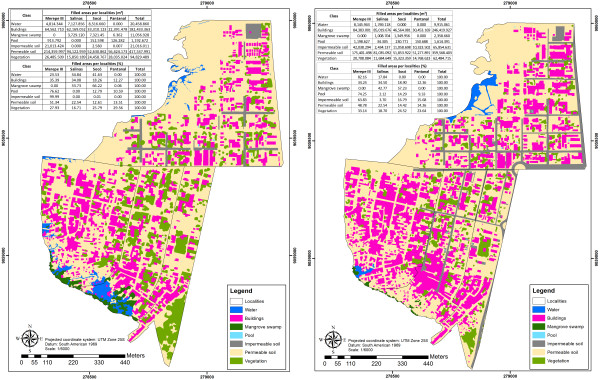
**Land use map for the area of epidemiological importance for schistosomiasis in Porto de Galinhas, Pernambuco - Brazil, 2006–2010.**

The reduction in the risk in Merepe III was due to the urban improvements that had been implemented there over the years. Pavement on the main streets made the ground impermeable (Figures 5 and 6), a protective factor against disease occurrences reducing the conditions that favor establishment of breeding sites for intermediate hosts [26], by impediment the aestivation process of *B. glabrata*, which is responsible for the resistance and survival through the dry season. Figures 3 and 3D show the shift of the spatial risk of schistosomiasis transmission from Merepe III to Salinas between 2000 and 2010, demonstrating that in 2000, Salinas presented a potential condition of risk, which ten years later had become the established reality.

The shifts in the area at risk of occurrences of schistosomiasis in Porto de Galinhas are directly related to the way in which the urban space has been occupied. Space has been expropriated and resized through social relationships of exclusion and improper appropriation, thereby imposing “privileged spaces” for tourists and insalubrious “peripheral spaces” for the natives [32]; these privileged spaces are located by the beach and it is represented by several five stars hotels and sophisticated restaurants. On the other hand, the native population is pushed to live in the peripheral spaces, close to the mangrove area, without any sanitation and exposed to the risk of being infected, especially in the rain season where the area is flooded. This is expressed as the high epidemiological risk for the latter population.

### Remote sensing

The environmental changes that took place between 2006 and 2010, as they’re shown by the satellite images, made it possible to quantify the process of occupation of this space, and added new information related to the formation of the present epidemiological structure for schistosomiasis transmission.

Figure 5 presents map-view images of Porto de Galinhas, showing the layout of the districts (Merepe III, Salinas, Socó and Pantanal) and the land occupation over the four-year period previously mentioned. Figure 6 illustrates land use per district, quantified according to class, thus enabling comparison and identification of the degree of variation per class over these years. It can be seen that there was expansion of the urban area (with construction of new buildings) towards the mangrove swamp areas in the localities of Salinas and Socó, and replacement of areas of vegetation by buildings in the localities of Socó and Pantanal. These maps clearly show reductions in the areas of mangrove swamp in Salinas (3,729 to 1,008 m^2^) and Socó (7,321 to 1,349 m^2^); reductions in the areas of vegetation in Socó (24,458 to 15,323 m^2^) and Pantanal (28,035 to 14,768 m^2^); and increases in the areas covered by buildings in all the districts, with changes of more than 30%. These data demonstrate the intensity of the occupation process on the periphery of Porto de Galinhas over the four-year period (Table 2) and highlight the invasion of ecotopes zoned for environmental preservation, such as mangrove swamps. These which were improperly landfilled lost their primary function of sheltering fish and crustaceans and were transformed into favorable areas for establishment of peridomestic foci of schistosomiasis transmission.

**Table 2:** **Percentage change in the areas occupied per class**, **between 2006 and 2010 in the districts of Porto de Galinhas**, **Pernambuco** - **Brazil**

**Land use areas**	**Change in use land areas/ Districts(%)**				**Total(%)**
	**Merepe III**	**Salinas**	**Socó**	**Pantanal**	
Water	69.20	−75.18	−100.00	0	−51.54
Buildings	30.70	36.76	39.79	36.00*	35.07
Mangrove swamp	0	−72.95	−81.56	−100.00	−78.67
Pool	31.17*	34.205	51.23*	19.33*	35.36
Impermeable soil	100.05	2.434,037**	428.531**	147.478,500**	213.35
Permeable soil	−18.17	−13.91*	−1.48	−9.12	−13.88
Vegetation	−21.81	cxx−26.28	−37.35	−47.32	−34.11

* p-value > 0.05 (i.e. without any statistically significant difference) – χ2 test.

** districts that presented null or almost null values for this class in 2006.

With regard to the high coverage of impermeable ground in Merepe III, there was an increase of 100% between 2006 and 2010 (21,013 to 42,038 m^2^), as it is shown in Figure 6. This area of impermeable ground consisted mainly of asphalting for the streets, which made it impossible for breeding sites for *B. glabrata* to exist, since these require earth and water. On the asphalted streets, snails could be found in the gutters and water drains (temporary breeding sites of *B. glabrata*), but did not resist the desiccation that occurs in this type of environment, since the asphalt made the mollusks’ process of aestivation impossible. The locality of Salinas is highlighted as an area with few paved areas, which favored the presence of breeding sites and maintenance of schistosomiasis cases. The kernel maps (Figures 3 and 4) show that the risk of disease occurrence and transmission were concentrated in the locality of Salinas in 2010.

Other factors that highlight the environmental degradation in the locality were the reductions in the areas of permeable ground and vegetation seen in all the districts and the increase in the area with pools, which can be correlated with the process of urbanization of the area (Table 2). The reduction of 51.54% in the total percentage of water (Figure 6 and Table 2) demonstrates the process of landfill and invasion of natural mangrove swamp areas. In Merepe III, an increase in the area occupied by estuarine waters and salinized water from the Ipojuca river was observed. This was probably due to the landfilled areas seen in Salinas (Figure 6).

The satellite images (Figure 5) and the land use maps (Figure 6) make it clear that there was an intense process of urbanization and improper invasion of the environment, with a significant reduction in the area covered by mangrove swamp from 11,057 m^2^ to 2,520 m^2^ (− 78%) in Salinas and Socó, within a scenario of street blocks presenting the greatest numbers of foci and human cases of the disease. It can be concluded that this type of environment is highly favorable for producing schistosomiasis, since over the course of these 10 years, Salinas was the only locality that had a significant increase (233%) in occurrences of *B.glabrata* foci, with a consequent maintenance of the rates of human infection.

## Conclusions

The increasing agglomeration of individuals within a limited area and within a chaotic process of occupation promoted an insalubrious structure that favored maintenance and dissemination of diseases such as schistosomiasis, with the potential for expansion to other regions and for exceeding the indicators for rural areas where the disease is historically endemic in northeast of Brazil. It can be concluded that identifying environmental changes related to establishment and maintenance of sanitary and biological factors that ensure perfect conditions for maintaining foci of schistosomiasis transmission could be measured by the remote-sensing technique, which is becoming a valuable tool to epidemiological studies. Special attention must be given to those changes, in a way to prevent the advance of schistosomiasis endemic areas to the coastline of Pernambuco State - Brazil.

By saying that, we are pointing out the need of an environmental monitoring using all available technology in order to detect environmental changes that could favor the increase of schitosomiasis. Furthermore, the development of predicting models for that mentioned disease is becoming a health urge because when it's based on malacological, epidemological and environmental data, as those ones presented in this paper - it may be possible to provide forthcoming scenarios of risks regarding schitosomiasis which is the next step our research.

## Abbreviations

*B. glabrata*: *Biomphalaria glabrata*; CPqAM: Aggeu Magalhães Research Center.

## Competing interests

The authors declare that they have no competing interests.

## Authors' contributions

ECSG: Conception of the project; data gathering, analysis and interpretation; writing the manuscript; and important critical review of the intellectual content. OBLN: Data gathering at the fieldwork stages and spatial analysis. JA: Conception of the project and statistical analysis on the data. HPS: Analysis on the satellite images. CSB: Conception and coordination of the project, critical review of the manuscript and final approval of the version to be published. All authors read and approved the final manuscript.

## Authors' information

ECSG: PhD Public Health and MSc in Animal Biology - Professor of parasitology at Universidade Feraral de Pernambuco – Brazil.

OBLN: Public Health Master's student at Centro de Pesquisas Aggeu Magalhães – Fiocruz – PE – Brazil.

JA: PhD and MSc in Computer Science – Professor of the Department of Statistics and Informatics – Universidade Federal Rural de Pernambuco – Brazil.

HPS: PhD in Soil Science and MSc in Remote Sensing – Professor and Coordinator of the Laboratory of GIS and Remote Sensing - GEOSERE - Universidade Federal Rural de Pernambuco – Brazil.

CSB: PhD Public Health and MSc in Biological Sciences – Head researcher of the department of parasitology and Coordinator of the Schistosomiasis Laboratory – CPqAM– Fiocruz – PE – Brazil.
